# Trusted Voices: Assessing Trusted Sources of Human Papillomavirus Vaccine Information Among a Sample of Hispanic Parents

**DOI:** 10.3390/vaccines13090917

**Published:** 2025-08-28

**Authors:** Alyssa A. Martinez, Surendranath S. Shastri, Gabriel A. Frietze

**Affiliations:** 1 College of Health Sciences, University of Texas at El Paso, El Paso, TX 79902, USA; aamartinez18@utep.edu; 2 Department of Health Disparities Research, Division of Cancer Prevention and Population Sciences, The University of Texas MD Anderson Cancer Center, Houston, TX 77030, USA; ssshastri@mdanderson.org; 3 Department of Pharmaceutical Sciences, School of Pharmacy, University of Texas at El Paso, El Paso, TX 79902, USA

**Keywords:** HPV vaccine, trusted sources of information, Hispanic, healthcare provider recommendations

## Abstract

Background/Objectives: Hispanics living in the United States have higher rates of diagnosis and mortality from certain kinds of cancers, including human papillomavirus (HPV)-related cancers. HPV vaccines can prevent 90% of HPV-associated cancers. Methods: The purpose of this study was to recruit a sample of Hispanic parents to investigate trusted sources of HPV vaccine information. An online survey was used to collect data from Hispanic parents who reported having children between the ages of 11 and 17. Results: Parents of children 11–17 years of age (*n* = 203, *M*_age_ = (38, *SD* = 6.97; female 85.1%) were included. The top five trusted sources of HPV vaccine information were medical doctors (95.1%), registered nurses (54.2%), the CDC (47.8%), the WHO (45.3%), and pharmacists (25.6%). The two least trusted sources were the president of the U.S. (7.9%) and religious leaders (3%). Hierarchical linear regression models revealed that HPV vaccine acceptance was associated with trusting registered nurses (*p* < 0.001) and the CDC (*p* = 0.026) in recommending the HPV vaccine. Importantly, the family-held belief that vaccines cause autism was strongly correlated with personal beliefs that vaccines cause autism (*r* = 0.58; *p* < 0.001). Conclusions: Findings from this study have clinical implications for the development of interventions and health communication strategies that leverage trusted sources of information including medical doctors and registered nurses to encourage preventive health behaviors. Additionally, our findings support that pharmacists should be included in these interventions as they are often an underused resource and are trusted by their patients for vaccine information.

## 1. Introduction

Human papillomavirus (HPV) is one of the most common sexually transmitted infections (STIs) and a leading cause of several cancers in both men and women [1]. Nearly all cases of cervical cancer in women are attributable to HPV, which also causes other reproductive cancers, including cancers of the vulva, vagina, penis, anus, rectum, and oropharynx [2]. In 2022, the World Health Organization (WHO) reported that HPV infection was responsible for approximately 660,000 new cases of cervical cancer and 350,000 deaths worldwide, making cervical cancer the fourth most common cancer among women [3]. In the United States, an estimated 37,800 HPV-associated cancers are diagnosed each year [2], with Hispanic women facing a 40% higher incidence of cervical cancer compared to non-Hispanic White women [4]. Furthermore, the Centers for Disease Control and Prevention (CDC) reports that Hispanic women experience higher mortality rates from cervical cancer than their non-Hispanic counterparts [5].

Currently, there are six HPV vaccines available which can prevent approximately 90% of HPV-associated cancers [6]. The HPV vaccine is approved for both females and males ages 11 to 45 [7], though it is most effective when administered prior to exposure to HPV [8]. Current CDC guidelines recommend that children ages 11–12 receive two doses administered 6–12 months apart; however, vaccination can begin as early as age 9. Children aged 9–14 years who receive two doses less than five months apart should receive a third dose [8]. Individuals aged 15–26 years are recommended to receive a three-dose series. Vaccination is not recommended for those older than 26, unless discussed otherwise with a healthcare provider (HCP) [8]. These guidelines are consistent with the WHO’s position on early vaccination, though global implementation varies widely, with some countries adopting a single-dose schedule to improve coverage in resource-limited settings [9].

Since its introduction, the HPV vaccine has contributed to global declines in HPV infection, pre-cancerous lesions of the cervix, cervical cancer, and genital warts, demonstrating its positive public health impact [10]. High rates of vaccination among both female and males can reduce the amount of HPV cases globally and improve reproductive cancer disparities among Hispanics in the United States. However, HPV vaccination rates dropped by 75% during the COVID-19 pandemic, largely due to disruptions in routine pediatric care, as many parents delayed or avoided scheduling their child’s regular healthcare appointments due the pandemic-related concerns [6]. In the United States, reductions in HPV vaccination were particularly pronounced, with one study reporting that HPV had the largest decline among adolescent vaccines [11]. Additionally, in a predominantly Hispanic border community, HPV vaccination rates dropped from 89.92% pre-pandemic to 69.59% during the pandemic [12]. Nationally, Hispanic adolescents (ages 9–17) have lower HPV vaccine initiation (34.4%) compared to non-Hispanic white adolescents (39.9%) [13]. According to Hernandez et al. (2024), commonly reported barriers to HPV vaccination among Hispanics include limited knowledge and awareness of HPV-related health risks, the absence of provider recommendations, and parental concerns that vaccination might encourage sexual promiscuity among adolescents [14]. Furthermore, in a systematic review conducted by Cangelosi et al. (2025) researchers found that barriers in Latin American countries included knowledge gaps such misconceptions about HPV transmission and vaccine safety [15].

Understanding both national and international epidemiology along with the challenges that influence vaccine acceptability, such as culture, is crucial for developing culturally competent public health messages that promote HPV vaccine uptake. There is a critical need for the development of culturally competent public health messages that are effective at promoting the HPV vaccine in Hispanic families, a population that may face unique barriers related to healthcare access, cultural beliefs, and trusted sources of health information. Our study will address this gap by providing data to drive the development of public health messages that promote HPV vaccination through the use of trusted sources of information (or trusted messengers of vaccine information).

### 1.1. Hispanic Culture and the Role of Family

Cancer outcomes are highly influenced by social, cultural, behavioral, and biological factors [16]. MA culture strongly values family cohesiveness. As a result, families tend to be large, multigenerational, and tight knit. Due to this nature of cohesiveness, it is imperative to increase vaccine acceptance among families. Receiving the HPV vaccine may be predicted by measuring the extent to which one’s family had positive or negative attitudes about vaccines while growing up and the level of risk that their family attributed to vaccines [17]. Thus, family perceptions of vaccines may be a key determinant of vaccine acceptance in MAs. Given that parents are the primary decision makers for childhood vaccinations, it is imperative to understand the factors associated with parental attitudes about vaccines. Additionally, parents often rely on medical doctors or other sources of health information when making informed vaccination decisions for their children; thus, it is important to investigate who parents trust the most for HPV vaccine recommendations.

### 1.2. Trusted Sources of Vaccine Information

HCP recommendations play a pivotal role in increasing acceptance of the HPV vaccine. In a review by Olson et al. (2020), which examined communication strategies to address parental hesitancy toward childhood vaccinations, HCPs were identified as the most influential source of vaccine information for parents [18]. Notably, the authors emphasized that who delivers the vaccine message may be just as important as the message itself. Oh et al. (2021) conducted a meta-analysis on HCP communication and HPV vaccine uptake [19]. The study found that HCP recommendations were associated with higher HPV vaccine initiation. Furthermore, HCP recommendations were also found to be associated with higher follow-through of the three-dose series for both females and males. The degree to which individuals trust vaccine information is related to who the messenger of the vaccine information is (e.g., medical doctor vs. social media). These findings highlight the need for identifying who MAs trust the most when receiving vaccine information and have implications for informing the development of vaccine promotion interventions and public health messages which include trustworthy messengers. The current study addressed this gap by identifying trustworthy messengers of HPV vaccine information.

### 1.3. Theoretical Framework

The Health Belief Model (HBM) [20] suggests that numerous factors (e.g., perceived susceptibility) predict preventive behaviors such as being vaccine uptake. Guided by the HBM, we have identified factors associated with accepting the HPV vaccine [17], the COVID-19 vaccine [21], and the influenza vaccine in MA adults [21,22]. The current study examines trusted sources of vaccine information while utilizing the HBM to examine theoretical predictors of HPV vaccine acceptance. Moreover, the current study investigates the role of family, which may be a unique cultural factor for MAs.

### 1.4. Purpose

This cross-sectional study aimed to gather early insights into which messengers are viewed as credible sources of HPV vaccine information by MA parents of children aged 11 to 17 years (*n* = 203). The urgency of this research is underscored by the sharp decline in routine pediatric vaccinations during the COVID-19 pandemic. Addressing this ongoing gap is critical, especially in MA communities, where the burden of HPV-related cancers remains disproportionately high. Examining trustworthy messengers (e.g., HCPs, family and friends, religious and community leaders, and health authorities) of HPV vaccine information may provide insight and the impetus for designing a future intervention testing trustworthy messengers of HPV vaccine information. The current project was guided by the HBM as our theoretical framework for understanding factors that contribute to vaccine acceptance.

### 1.5. Aims and Hypotheses

The primary aim of this project was to identify who parents trust for HPV vaccine recommendations. The secondary aim of this project was to examine factors that are associated with HPV vaccination. It is hypothesized that the most trusted source of HPV vaccine information will be healthcare providers. That is, medical doctors (MDs) will be perceived as the most trusted source of HPV vaccine information. In addition, it is hypothesized that the HBM constructs (perceived susceptibility, severity, benefits, and barriers) will predict vaccine acceptance.

## 2. Materials and Methods

### 2.1. Study Design

A cross-sectional survey designed for an inventory assessment of HPV-related items was deployed in a Hispanic majority sample between October 2022 and January 2023, following the STROBE reporting guidelines (See Supplementary Files S1).

### 2.2. Setting

The study was conducted in El Paso, Texas, a United States/Mexico border city with a predominately Hispanic population.

### 2.3. Participants

Parents with children eligible for the HPV vaccine (ages 11–17) were recruited to complete a 30-min online survey and were compensated with a USD 15 electronic gift card for their participation. The survey assessed trustworthy messengers of HPV vaccine information and social, cultural, and behavioral correlates of accepting the HPV vaccine. Participants were recruited using Facebook geotargeted advertisements. A power analysis using G*Power 3 [23] indicated that 202 participants would be needed to provide a 95% chance of detecting a small effect (*r* = 0.25) with alpha set to 0.05. The estimated effect size was derived from a meta-analysis which included correlates of vaccine acceptance which ranged from 0.25 to 0.51 [24]. The smallest weighted average effect size reported in the meta-analysis was used to compute power. Inclusion criteria for our study included (1) being age 18 or older; (2) living in El Paso, Texas; (3) being a parent of a child 11–17 years of age; and (4) speaking either English or Spanish.

### 2.4. Measures

A 30-min survey was administered to parents to assess basic demographics, trusted sources of HPV information, familial attitudes about vaccines, and factors from the HBM. See Table 1 for measurement details.

### 2.5. Data Sources/Measurement

The survey was administered via QuestionPro which can be used on a computer, cellphone, or tablet. Facebook’s Geotargeting features were utilized to target individuals who live in El Paso and are over the age of 18.

### 2.6. Bias Mitigation

The “prevent ballot stuffing” option via QuestionPro was utilized to prevent participants from completing the survey more than once. Additionally, the El Paso Vaccine Community Advisory Board (EPV-CAB) was developed to provide feedback on our surveys and recruitment approaches, ensuring that we included culturally and linguistically sensitive assessments. The EPV-CAB conducted quarterly meetings to ensure input from community stakeholders with diverse backgrounds, including a director from a local immunization clinic, a registered nurse, a licensed vocational nurse working at a local youth educational program, a community needs coordinator from the Texas Department State Health Services (DSHS) public health, and an operations manager from the El Paso Pediatric Association.

### 2.7. Approach to Analysis

Descriptive statistics delineated participant characteristics. Means and standard deviations were examined for continuous variables, and percentages were examined for categorical variables. Percentages from a select-all-that-apply-style item were calculated and used as a preliminary index of trustworthy messengers to promote the HPV vaccine. Items on a Likert-type scale assessed the level of trust for each HPV vaccine messenger. Multiple imputation was not used for missing data given that less than 5% of the data was missing. Correlation analyses examined the association between individual and family beliefs about vaccines. A Bonferroni correction was applied to account for multiplicity in testing (α/10 = 0.005). Two hierarchical linear regression models examined predictors of HPV vaccine likelihood, which was a continuous variable. Moreover, two hierarchical logistic regression models examined predictors of ever vaccinating child with the HPV vaccine (Yes/No), which was a dichotomous variable. In each of the models, sociodemographic variables (age, primary language, household income, and gender identity) were entered in step 1, and all other variables were entered in step 2. Model 1: A hierarchical linear regression analysis was performed to predict HPV vaccine likelihood based on the top five trusted sources of information after controlling for age, primary language, household income, and gender identity. Model 2: Hierarchical logistic regression was performed to assess the impact of the top five trusted sources of information on ever vaccinating child with the HPV vaccine (Yes/No) after controlling for age, primary language, household income, and gender identity. Model 3: A hierarchical linear regression analysis was performed to predict HPV vaccine likelihood using five factors from the HBM (perceived safety, perceived effectiveness, perceived harm, perceived susceptibility, and perceived barriers) after controlling for age, primary language, household income, and gender identity. Model 4: Hierarchical logistic regression was performed to predict ever vaccinating child with the HPV vaccine (Yes/No), using factors from the HBM after controlling for age, primary language, household income, and gender identity.

## 3. Results

### Participant Characteristics

A total of 203 Hispanic adults (*M*_age_ = 38, *SD* = 6.97) who identified predominately as female (85.1%) were included in the study. Twenty-two participants were excluded for not identifying as Hispanic. Some 74% (*n* = 152) of the sample completed the survey in English, and 25.1% (*n* = 51) completed the survey in Spanish. Spanish was the primary language spoken at home in 43.4% (*n* = 86) of the sample. More than a third of the sample (39.4%) of the sample reported that their annual income was below USD 40,000, and 62.6% of the sample reported that their annual income was below USD 60,000. Most of the sample reported to be married (57.1%, *n* = 116). The most frequent religious affiliations that participants identified were Catholicism (50.7%, *n* = 103) and Christianity (25.1%, *n* = 51). Over a third (39%, *n* = 78) of the participants reported having three or more children, and only 68% (*n* = 138) of the sample reported having vaccinated at least one of their children with the HPV vaccine. See Table 2 for demographic and background information.

The top five credible and trustworthy sources of HPV vaccine information (as indexed by the “select all that apply” items) were medical doctors (95.1%), registered nurses (54%), the Centers for Disease Control [and Prevention] (47.8%), the World Health Organization (45.3%), and pharmacists (25.6%). Religious leaders (3%) were reported to be the least trustworthy source of HPV vaccine information. See Table 3 for trusted sources of HPV vaccine information. The top five credible and trustworthy sources of HPV vaccine information (as indexed by the Likert-type scale items) were medical doctors (*M* = 4.05, *SD* = 1.33), registered nurses (*M* = 4.00, *SD* = 1.08), the Centers for Disease Control and Prevention (*M* = 3.94, *SD* = 0.99), the World Health Organization (*M* = 3.95, *SD* = 1.00), and pharmacists (*M* = 3.50, *SD* = 1.07).

Correlations ranged from −0.27 to 0.73 (see Table 4). Correlational analyses revealed that individual vaccine beliefs were strongly associated with family vaccine beliefs. The strongest correlation that emerged was between having a family with negative feelings about vaccines while growing up and their family believing that vaccines can cause serious illness (*r* = 0.73; *p* < 0.001). Importantly, the belief that vaccines cause autism was strongly correlated with one’s family’s belief that vaccines cause autism (*r* = 0.58; *p* < 0.001; see Table 4).

Model 1 included a hierarchical linear regression analysis predicting HPV vaccine likelihood based on five trustworthy sources of information: medical doctors (MDs), registered nurses (RNs), the Centers for Disease Control and Prevention (CDC), the World Health Organization (WHO), and pharmacists. Sociodemographic variables including age, language, household income, and gender identity were included in step 1 of the model, and the five trustworthy sources of information were entered in step 2 of the model. Preliminary analyses were conducted to ensure no serious violations of the assumptions of normality, linearity, multicollinearity, and homoscedasticity. After controlling for sociodemographic variables, the results indicate that trust in RNs (beta = 0.35, *t* = 3.36, *p* < 0.001) and the CDC (beta = 0.28, *t* = 2.24, *p* = 0.026) significantly predicted HPV vaccine likelihood (*F* (9, 178) = 7.2 *p* < 0.001), with an *R*^2^ of 0.035 in step 1 and an adjusted *R*^2^ of 0.277 in step 2. More specifically, for every one-unit change in trusting RNs, there was a 0.35 increase in HPV vaccine likelihood, holding all other variables constant. In addition, for every one-unit change in trusting the CDC, there was a 0.28 increase in HPV vaccine likelihood, holding all other variables constant. See Table 5 for linear regression predicting HPV vaccine likelihood.

Model 2 included a hierarchical logistic regression assessing the impact of the five trusted sources of information on ever vaccinating child with the HPV vaccine (Yes/No). Sociodemographic variables including age, language, household income, and gender identity were included in step 1 of the model, and the five trustworthy sources of information were entered in step 2 of the model. The full model containing all predictors was statistically significant, *Χ*^2^ (5, *n* = 203) = 14.89, *p* = 0.011, indicating the model was able to distinguish between parents who vaccinated and did not vaccinate their child with the HPV vaccine. The model as a whole explained between 0.14% (Cox and Snell R square) and 0.20% (Nagelkerke R squared) of the variance in vaccine uptake and correctly classified 75.3% of cases. However, none of the factors emerged as statistically significant predictors of ever vaccinating child with the HPV vaccine (Yes/No).

Model 3 included a hierarchical linear regression analysis predicting HPV vaccine likelihood based on the five factors from the HBM. Sociodemographic variables were included in step 1 of the model, and the five factors from the HBM were entered in step 2 of the model. Preliminary analyses were conducted to ensure no serious violations of the assumptions of normality, linearity, multicollinearity, and homoscedasticity. After controlling for sociodemographic variables, the results indicate that perceived effectiveness (beta = 0.50, *t* = 3.86, *p* < 0.001) and perceived harm (beta = −0.30, *t* = −2.21, *p* = 0.029), significantly predicted HPV vaccine likelihood (*F* (9, 176) = 9.34, *p* < 0.001), with an *R*^2^ of 0.04 in step 1 and an adjusted *R*^2^ of 0.30 in step 2. More specifically, for every one-unit change in perceived effectiveness, there was a 0.50 increase in HPV vaccine likelihood, holding all other variables constant. In addition, for every one-unit change in perceived harm, there was a 0.30 decrease in HPV vaccine likelihood, holding all other variables constant. See Table 6 for linear regression predicting HPV vaccine likelihood from the HBM.

Model 4 included a hierarchical logistic regression was performed to examine the impact of the HBM on ever vaccinating child with the HPV vaccine (Yes/No). Age, language, household income, and gender identity were included in step 1 of the model, and the five factors from the HBM were entered in step 2 of the model. The full model containing all predictors was statistically significant, *Χ*^2^ (4, *n* = 203) = 16.28, *p* = 0.012, indicating the model was able to distinguish between parents who vaccinates and did not vaccinate their child with the HPV vaccine. The model as a whole explained between 0.15% (Cox and Snell R square) and 0.22% (Nagelkerke R squared) of the variance in HPV vaccine uptake and correctly classified 78.3% of cases. However, only perceived harm (beta = −0.73, *p* = 0.031) emerged as a statistically significant predictor of having ever vaccinated child with the HPV vaccine.

## 4. Discussion

The disparities in HPV vaccination and cervical cancer incidence and mortality among Hispanics underscore the critical importance of developing effective HPV vaccination programs and the need to tailor prevention strategies to populations at greatest risk. The current study highlights the critical role of credible and trusted sources of health information, such as MDs and other HCPs in promoting HPV vaccination. The degree of trust in vaccine information is closely related to the messenger delivering the information. In this study, the top five trusted sources of HPV vaccine information were MDs, RNs, the CDC, the WHO, and PharmDs. These findings align with a seminal review by Brewer and Fazekas (2007) which found that both parents and young adults were more likely to accept the HPV vaccine when it was recommended by a healthcare provider [30]. Similarly, recent research by Lucero et al. (2025) reported that HCP communication and recommendations significantly influenced HPV vaccine initiation in a population of Central American immigrant parents; however, less than 28% of participants reported receiving an HPV vaccine recommendation [31]. Past findings along with the findings from the current study have significant public health and clinical implications, particularly in reinforcing the importance of HCPs consistently recommending the HPV vaccine to their patients. Moreover, this study supports that pharmacists should have an active role in recommending the HPV vaccine given that they are trusted by their patients for vaccine information. This study also provides valuable insight for developing public health messaging that leverages trusted sources to increase vaccine uptake in medically underserved communities, such as El Paso, Texas, where access to providers may be limited.

Furthermore, the findings from this study suggest that religious leaders may not be effective messengers for promoting vaccine information among MAs, as they were identified as the least trusted source, with only 2.7% of participants reporting trust in them. Current research on the role of religious leaders in vaccine promotion has yielded mixed results. For example, Hicken et al. (2024) found that in areas where medical professionals already endorsed vaccines, religious leaders’ support influenced vaccine uptake in only one out of six countries studied, highlighting that medical practitioners remain the most effective advocates for vaccination [32]. Similarly, Purvis et al. (2021) found that just 18.86% of individuals reported being “very likely” to seek vaccine information from religious leaders [33], and Seyd et al. (2025) reported that faith leaders and national government were among the least trusted sources of COVID-19 information in a British population [34]. Interestingly, other findings indicate a potential indirect role for religion in vaccine communication. A study by Chu et al. (2021) found that physicians who disclosed their religious identity were more likely to earn the trust of vaccine-hesitant Christians [35]. These insights, alongside the current study’s findings, suggest that while religious leaders themselves may not be the most persuasive vaccine advocates, trusted healthcare providers (e.g., MDs, RNs) may enhance the effectiveness of their recommendations by acknowledging shared values or identities, such as religion, when engaging with hesitant populations.

Another important finding that emerged is that individual vaccine beliefs were strongly associated with family vaccine beliefs. For example, the belief that vaccines cause autism was strongly correlated with one’s family’s belief that vaccines cause autism. Similar results are reported by Frietze et al. (2023a) using the same assessment tool [17]. This finding highlights the importance of developing family-based interventions for promoting the HPV vaccine and reducing misconceptions associated with vaccines at the family level.

Finally, factors from the HBM emerged as predictors of likelihood to accept the HPV vaccine and for parents having ever vaccinated their child. In a literature review among studies that pertained to African American and Latino parents, Galbraith et al. (2016) found that vaccine safety, effectiveness, and provider recommendations were positively associated with uptake and acceptability [36]. Consistent with the HBM, Zampetakis and Melas (2021) report that individuals who perceive high levels of severity of infection will express higher intentions to vaccinate [37]. These findings carry significance for the use of theoretical frameworks to guide the development of public health interventions and public health messages aimed at promoting vaccines.

This study has several strengths, including the unique population of Hispanic parents recruited from non-clinic settings who may not have access to routine care. Our focus on Hispanic parents allows for understanding factors that may be specific to this population and that influence who individuals trust for vaccine information. Another strength is that we evaluated levels of trust associated with a large number of sources of information ranging from HCPs to religious leaders, yielding insight into who individuals trust the most for vaccine information. Finally, an additional strength that emerged is the inclusion of the HBM to guide our understanding of the factors that contribute to vaccine intentions.

Despite the strengths of this study, a number of limitations exist. Although focusing on a Hispanic population provides important findings about who individuals trust for vaccine information, these findings may not be generalizable to other ethnic groups. Additionally, this study was conducted online and utilized online recruitment strategies, which may have contributed to issues accessing the survey. Another limitation of the study is that it utilized a cross-sectional design which cannot establish causal relationships. Future studies should utilize an experimental design to identify whether trusted sources of information truly cause differences in vaccine acceptance.

## 5. Conclusions

This study underscores the importance of trusted messengers in shaping HPV vaccine acceptance among Hispanic parents. Consistent with our hypothesis, medical doctors and registered nurses emerged as the most credible sources of HPV vaccine information, alongside institutional authorities such as the CDC and WHO. Importantly, pharmacists also surfaced as underutilized yet trusted sources, highlighting an opportunity to expand their role in vaccine promotion, especially in medically underserved communities with limited access to physicians. Conversely, religious and political figures were identified as the least trusted messengers, suggesting that public health interventions relying on these voices are unlikely to be effective within this population.

Beyond identifying trusted sources, our findings demonstrate that parental vaccine acceptance is influenced by both individual and family beliefs, with misconceptions such as the belief that vaccines cause autism. This reinforces the need for culturally sensitive, family-centered approaches that address intergenerational attitudes and misinformation. Guided by the HBM, we found that perceived effectiveness and perceived harm were key predictors of vaccine likelihood, underscoring the relevance of theoretical frameworks in understanding and addressing parental decision making. These results have several clinical and public health implications. Interventions aimed at increasing HPV vaccination rates in Hispanic communities should (1) prioritize HCPs, pharmacists, and public health authorities as frontline messengers, (2) integrate family-level education to counteract misinformation and strengthen collective acceptance of vaccines, and (3) employ theoretical models such as the HBM to design and evaluate communication strategies. Furthermore, future research should explore experimental designs to establish causal relationships and further refine intervention strategies. By leveraging trusted voices and addressing culturally rooted beliefs, public health interventions can advance HPV vaccination uptake and reduce HPV-associated cancer disparities among Hispanic populations.

## Figures and Tables

**Table 1:** Assessment tools.

Variable Name	Description	Sample Item(s)
Basic Demographics Survey	Fifteen items assessing basic demographics such as age, gender, ethnicity, primary language spoken at home (English or Spanish) and related information (see Supplementary Materials).	“What is your age in years?” “What is your primary language spoken at home?”
Background Questionnaire	Twenty-three items assessing parental status, who has provided vaccine recommendations, household size (as a proxy of multigenerational households), and items related to vaccinating one’s own children (see Supplementary Materials).	“Who has provided you with recommendations or guidance about whether YOUR CHILD should receive the HPV vaccine?” (Select all the apply). Fourteen response options were provided including a “pediatrician”, “family doctor”, “pharmacist”, and a “school nurse”. Responses were coded as 1 = yes and 0 = no. Participants could select more than one response option, and a composite was created by adding up responses. Scores range from 0 to 13.
Independent Variables		
Trustworthy HPV Informational Sources	A 13-item questionnaire adapted from Frietze et al. (2023b) assessed trustworthy HPV informational sources [21] (see Supplementary Materials).	“I would trust information about the HPV vaccine if the information was provided from a Medical Doctor (MD).” Response options range from (1) Strongly disagree, to (5) Strongly agree.
Perceived Severity	A single item adapted from Katz et al. (2011) assessed perceived severity of HPV [25].	“How severe do you think genital HPV infection is for yourself?” Response options range from (1) Strongly disagree, to (5) Strongly agree.
Perceived Safety	Three items adapted from Brabin et al. (2006) assessed perceived safety of the HPV vaccine [26].	“I worry about the short-term side effects of the HPV vaccine.” “I worry that the HPV vaccine might negatively affect my body.” “I worry that the HPV vaccine might have unknown long-term side effects.” Response options range from (1) Strongly disagree, to (5) Strongly agree. Response options were reverse-coded for all three questions so that higher scores indicate greater perceived safety of the HPV vaccine. A composite score was created by averaging the three items.
Perceived Effectiveness	Three items adapted from Brabin et al. (2006) assessed perceived effectiveness of the HPV vaccine [26].	“I believe the HPV vaccine is effective in preventing genital HPV (e.g., vaginal, penile, or anal).” “I believe the HPV vaccine works in preventing genital HPV (e.g., vaginal, penile, or anal).” “I believe if I get the HPV vaccine, I will be less likely to get genital HPV (e.g., vaginal, penile, or anal).” Response options range from (1) Strongly disagree, to (5) Strongly agree. Higher scores indicate greater perceived effectiveness of the HPV vaccine.
Perceived Harm	Three items representing a subscale of the validated Carolina HPV Immunization Attitudes and Beliefs Scale (CHIAS) assessed perceived harm [27].	“I think the HPV vaccine may cause health problems in the future.” “I think the HPV vaccine is unsafe.” “I think the HPV vaccine might cause short-term problems like fever or discomfort.” Response options range from (1) Strongly disagree, to (5) Strongly agree. A composite score was created by averaging the three items. Previous studies have demonstrated high re-test reliability estimates (0.73–0.80) [27,28].
Attention	A single item served as an attention check. Failure to respond correctly to this item results in the exclusion of the subject’s data from analysis.	“If you are paying attention, please select “Blue” for the following response.” Response options included: (1) Red, (2) Green, (3) Blue, and (4) Yellow. Failure to respond correctly to this item results in the exclusion of the subject’s data from analysis.
Individual and Family Beliefs about Vaccines	Ten items from Frietze et al. (2020) assessed individual and family beliefs about vaccines [29].	“My family believes vaccines cause Autism”. Response options range from (1) Strongly disagree, to (5) Strongly agree.
Pandemic Barriers Towards Child Healthcare	Three items developed by the research team assessed barriers towards getting the HPV vaccine (see Supplementary Materials).	“The ongoing COVID-19 pandemic has been a barrier towards vaccinating my child”, “The ongoing COVID-19 pandemic has prevented me from taking my child to their pediatrician for their wellness check-ups”, “I have reduced the amount of times that a take my child to their pediatrician due to the COVID-19 pandemic.” Response options range from (1) Strongly disagree, to (5) Strongly agree. A composite score was created by averaging the three items.
Dependent Variables		
Ever vaccinated child with the HPV vaccine (Yes/No)	A single item adapted from Frietze et al. (2023a) assessed if parents have ever vaccinated their children with the HPV vaccine [17].	“Have any of your children received the Human Papillomavirus (HPV) vaccine?” Response options included: 0 = No, 1 = Yes, 2 = Prefer not to answer. Prefer not to answer responses were recoded as a missing value for purposes of analysis.
HPV Vaccine Likelihood (Self)	A single item developed by the research team assessed participants likelihood to get vaccinated with the HPV vaccine if eligible.	“How likely are you to get vaccinated with the HPV vaccine if you are eligible?” Response options range from (1) Extremely unlikely to (5) Extremely likely. One of the response options included the following: “I have already been vaccinated.” The latter response option was recoded as a missing value for purposes of analysis.

**Table 2:** Demographic and background information (*N* = 203).

Variable	Total Responses	Percentage of Responses
Ethnicity		
Hispanic	203	100%
Primary Language		
English	111	54.7%
Spanish	86	42.4%
Other	1	0.5%
Missing	5	2.5%
Language that survey was completed in		
English	152	74.9%
Spanish	51	25.1%
Household income		
Less than USD 5000	5	2.5%
USD 5001–USD 20,000	20	9.9%
USD 20,001–USD 40,000	55	27.1%
USD 40,001–USD 60,000	47	23.2%
USD 60,001–USD 80,000	34	16.7%
USD 80,001–USD 100,000	21	10.3%
USD 100,001 or more	17	8.4%
Do not know	2	1%
Prefer not to answer	2	1%
How many children do you have?		
1	47	23.2%
2	75	36.9%
3	44	21.7%
4	21	10.3%
5	9	4.4%
More than 5	4	2%
Missing	3	1.5%
Have any of your children ever received the HPV Vaccine?		
Yes	138	68%
No	54	26.6%
Prefer not to answer	2	1%
Unsure	7	3.4%
Missing	2	1%

**Table 3:** Trusted sources of HPV information (*N* = 203).

If You Were Receiving Information About HPV and the HPV Vaccine, Who Would You Believe Is the Most Credible and Trustworthy:	*n*	Percentage
Medical doctor (MD)	193	95.1%
Registered nurse (RN)	110	54.2%
Centers for Disease Control and Prevention	97	47.8%
World Health Organization (WHO)	92	45.3%
Pharmacist (PharmD)	52	25.6%
Dr. Anthony Fauci, Chief Medical Advisor of the President of the United States	55	27.1%
School nurse	47	23.2%
Community health worker or promotor(a) de salud	41	20.2%
Family/friends	21	10.3%
Community leader from El Paso (e.g., Mayor Oscar Leeser)	17	8.4%
The President of the United States	16	7.9%
Religious leader (e.g., a priest, pastor, or other)	6	3%

Note: Item is a “select all that apply” response; response options do not total to 100%.

**Table 4:** Individual and family beliefs about vaccines.

	1	2	3	4	5	6	7	8	9	10
1. I had negative feelings about vaccines while I was growing up.	-	-	-	-	-	-	-	-	-	-
2. I believe vaccines can cause serious illness.	0.65 **	-	-	-	-	-	-	-	-	-
3. I believe vaccines are safe and effective.	−0.35 **	−0.46 **	-	-	-	-	-	-	-	-
4. I believe everyone should be immunized against serious diseases.	−0.41 **	−0.49 **	0.52 **	-	-	-	-	-	-	-
5. I believe vaccines cause autism.	0.37 **	0.44 **	−0.42 **	−0.35 **	-	-	-	-	-	-
6. My family had negative feelings about vaccines while I was growing up.	0.60 **	0.46 **	−0.33 **	−0.38 **	0.30 **	-	-	-	-	-
7. My family believes vaccines can cause serious illness.	0.49 **	0.55 **	−0.41 **	−0.45 **	0.44 **	0.73 **	-	-	-	-
8. My family believes vaccines are safe and effective.	−0.34 **	−0.42 **	0.43 **	0.47 **	−0.27 **	−0.52 **	−0.57 **	-	-	-
9. My family believes everyone should be immunized against serious diseases.	−0.46 **	−0.55 **	0.47 **	0.60 **	−0.34 **	−0.59 **	−0.63 **	0.68 **	-	-
10. My family believes vaccines cause autism	0.37 **	0.43 **	−0.36 **	−0.46 **	0.58 **	0.45 **	0.55 **	−0.44 **	−0.50 **	-
Mean (SD)	2.05 (1.03)	2.23 (1.10)	4.19 (0.89)	4.26 (0.81)	2.02 (1.00)	1.89 (1.00)	2.05 (1.06)	4.01 (0.99)	4.11 (0.42)	2.06 (1.01)
*n*	201	201	202	202	202	202	202	202	202	202

Note: Correlations are reported using Pearson’s correlation coefficient (two-tailed). ** *p* < 0.001.

**Table 5:** Hierarchical linear regression predicting HPV vaccine likelihood (*n* = 199).

Variables	*B*	*SE B*	β	95% Confidence Intervals		*p*
				Lower	Upper	
Step 1						
Constant	3.634	0.740		2.173	5.096	<0.001
Age	−0.002	0.014	−0.009	−0.029	0.025	0.909
Primary Language	−0.009	0.185	−0.004	−0.373	0.356	0.963
Household Income	−0.110	0.057	−0.145	−0.224	−0.003	0.056
Gender Identity	0.387	0.250	0.117	−0.107	0.881	0.124
Step 2						
Constant	0.765	0.771		0.757	2.286	0.323
Age	−0.001	0.012	−0.005	−0.026	0.024	0.936
Primary Language	−0.071	0.167	−0.007	−0.348	0.313	0.917
Household Income	−0.118	0.051	−0.155	−0.219	−0.018	0.022
Gender Identity	0.278	0.224	0.084	−0.164	0.720	0.217
Medical Doctors (MDs)	0.014	0.075	0.015	−0.135	0.163	0.853
Registered Nurses (RNs)	0.346	0.103	0.299	0.143	0.550	<0.001
Centers for Disease and Control (CDC)	0.279	0.124	0.220	0.033	0.524	0.026
World Health Organization (WHO)	0.157	0.125	0.124	−0.089	0.403	0.210
Pharmacists	−0.015	0.096	−0.013	−0.204	0.175	0.878

Note: Higher scores for the dependent variable indicate higher likelihood to vaccinate self if eligible and not already vaccinated on a 5-point scale. Sample size is less than 203 due to missing data. *R*^2^ = 0.035 for step 1; *R*^2^_Adjusted_ = 0.238 for step 2.

**Table 6:** Hierarchical linear regression predicting HPV vaccine likelihood from HBM (*n* = 177).

Variables	*B*	*SE B*	β	95% Confidence Intervals		*p*
				Lower	Upper	
Step 1						
Constant	3.770	0.747		2.295	5.244	<0.001
Age	−0.004	0.014	−0.022	−0.031	0.023	0.772
Primary Language	−0.027	0.185	−0.011	−0.392	0.337	0.882
Household Income	−0.109	0.057	−0.144	−0.222	−0.004	0.059
Gender Identity	0.382	0.254	0.144	−0.118	0.883	0.133
Step 2						
Constant	2.273	1.155		0.008	4.554	0.051
Age	−0.014	0.012	−0.077	−0.037	0.009	0.233
Primary Language	0.000	0.164	−0.000	−0.325	0.324	0.999
Household Income	−0.071	0.049	−0.094	−0.168	0.025	0.144
Gender Identity	0.194	0.222	0.058	−0.244	0.632	0.383
Perceived safety	0.191	0.098	0.163	0.003	0.384	0.053
Perceived effectiveness	0.500	0.130	0.294	0.244	0.756	<0.001
Perceived harm	−0.300	0.136	−0.198	−0.568	−0.032	0.029
Perceived severity	0.087	0.063	0.091	−0.037	0.210	0.170
Perceived barriers	0.013	0.085	0.011	−0.155	0.182	0.876

Note: Sample size is less than 203 due to missing data. *R*^2^ = 0.035 for step 1; *R*^2^_Adjusted_ = 0.299 for step 2.

## Data Availability

The dataset supporting the conclusions of this article is available at Mendeley repository.

## Supplementary Materials

The following supporting information can be downloaded at: https://www.mdpi.com/article/10.3390/vaccines13090917/s1, Files S1: STROBE checklist.

## Abbreviations

The following abbreviations are used in this manuscript:

HPV	Human papillomavirus
MD	Medical doctor
RN	Registered nurse
CDC	Centers for Disease Control
WHO	World Health Organization
PharmD	Pharmacist
